# The Predictive Diagnostic Value of Serial Daily Bedside Ultrasonography for Severe Dengue in Indonesian Adults

**DOI:** 10.1371/journal.pntd.0002277

**Published:** 2013-06-13

**Authors:** Meta Michels, Uun Sumardi, Quirijn de Mast, Hadi Jusuf, Mita Puspita, Intan Mauli Warma Dewi, Sylvia Sinarta, Bachti Alisjahbana, André J. A. M. van der Ven

**Affiliations:** 1 Department of General Internal Medicine, Radboud University Nijmegen Medical Centre, Nijmegen, The Netherlands; 2 Department of Internal Medicine, Faculty of Medicine, University of Padjadjaran, Bandung, Indonesia; Universidade de São Paulo, Brazil

## Abstract

**Background:**

Identification of dengue patients at risk for progressing to severe disease is difficult. Significant plasma leakage is a hallmark of severe dengue infection which can suddenly lead to hypovolemic shock around the time of defervescence. We hypothesized that the detection of subclinical plasma leakage may identify those at risk for severe dengue. The aim of the study was to determine the predictive diagnostic value of serial ultrasonography for severe dengue.

**Methodology/Principal Findings:**

Daily bedside ultrasounds were performed with a handheld ultrasound device in a prospective cohort of adult Indonesians with dengue. Timing, localization and relation to dengue severity of the ultrasonography findings were determined, as well as the relation with serial hematocrit and albumin values. The severity of dengue was retrospectively determined by WHO 2009 criteria. A total of 66 patients with proven dengue infection were included in the study of whom 11 developed severe dengue. Presence of subclinical plasma leakage at enrollment had a positive predictive value of 35% and a negative predictive value of 90% for severe dengue. At enrollment, 55% of severe dengue cases already had subclinical plasma leakage, which increased to 91% during the subsequent days. Gallbladder wall edema was more pronounced in severe than in non-severe dengue patients and often preceded ascites/pleural effusion. Serial hematocrit and albumin measurements failed to identify plasma leakage and patients at risk for severe dengue.

**Conclusions/Significance:**

Serial ultrasonography, in contrast to existing markers such as hematocrit, may better identify patients at risk for development of severe dengue. Patients with evidence of subclinical plasma leakage and/or an edematous gallbladder wall by ultrasonography merit intensive monitoring for development of complications.

## Author Summary

Dengue virus infection ranges from a mild febrile illness to severe illness. Severe dengue is mainly characterized by transient plasma leakage, which may lead to a sudden onset of shock around the time of defervescence. Severe bleeding and organ impairment are less common features of severe dengue. In clinical practice it is difficult to predict which dengue patient will develop severe complications. Commonly used laboratory markers indicating plasma leakage—such as hematocrit—are rarely of clinical benefit. In contrast, ultrasonography can directly visualize plasma leakage. Because ultrasonography is not routinely used due to financial and logistical limitations, we used a more affordable handheld ultrasound device for daily bedside follow up, which had similar results as conventional ultrasonography. This study shows that a substantial proportion of mildly ill patients already had small amounts of plasma leakage before severe complications developed and—most importantly—that these patients had an increased risk for progression to shock compared to patients without plasma leakage. We conclude that ultrasonography can help to identify dengue patients at risk for shock and that patients with ultrasonographic evidence of plasma leakage should be monitored more carefully for circulatory status to timely recognize and possibly prevent shock.

## Introduction

Dengue virus infection is the most rapidly spreading mosquito-borne viral disease in the world and has the highest burden in often resource poor (sub)tropical countries. Symptomatic dengue patients usually present with a self-limiting febrile illness, called dengue fever. However, a proportion of dengue patients develops severe complications around the time of defervescence.

Transient plasma leakage into serosal cavities, which may progress to life-threatening hypovolemic shock, is a hallmark of severe dengue [1], [2]. In the former World Health Organization (WHO) classification (1997), severe dengue characterized by fever, thrombocytopenia, plasma leakage and hemorrhagic tendency, is referred to as dengue hemorrhagic fever (DHF) and, when accompanied by circulatory failure, as dengue shock syndrome (DSS) (table 1) [3]. The most recent WHO classification (2009) defines severe dengue infection in case plasma leakage leading to shock or respiratory failure, severe bleeding or severe organ failure is present [4] (table 1). However, in clinical practice severe bleeding and severe organ failure are relatively uncommon and rarely occur without plasma leakage [5], [6]. Hence, plasma leakage is an essential element of severe dengue, regardless whether the WHO 1997 or 2009 classification is used.

**Table 1:** Criteria for severe dengue according to World Health Organization guidelines 1997 and 2009.

Criteria WHO 1997	Criteria WHO 2009
**DHF**	
Dengue with all of the following 4 components:	Dengue with any of the following:
1. Fever	1. Severe plasma leakage leading to shock
2. Hemorrhagic tendency	or respiratory distress
3. Thrombocytopenia (<100×10^9^ cells/L)	2. Severe bleeding as evaluated by clinicians
4. Plasma leakage:	3. Severe organ failure
a. Pleural effusion or ascites and/or	a. Liver (ASAT, ALAT >1000 U/L)
b. Minimum 20% hematocrit change and/or	b. Central nervous system: impaired consciousness
c. High hematocrit for population/age/sex and/or	c. Heart and other organs
d. Low serum protein	
**DSS**	
DHF and circulatory failure	

Abbreviations: WHO: World Health Organization; DHF: dengue hemorrhagic fever; DSS: dengue shock syndrome; ALAT: alanine aminotransferase; ASAT: aspartate aminotransferase.

One of the most important challenges for clinicians caring for dengue patients is to identify those patients who will progress to severe disease. Currently, no accurate biomarkers are available and prediction of significant plasma leakage is difficult in clinical practice. Serial hematocrit values are frequently used to detect hemoconcentration as a sign of plasma leakage, but are hard to interpreted due to individual variations, absence of baseline hematocrit values, intravenous fluid administration and bleeding. Monitoring for pleural effusion and/or ascites - both predilection sites of plasma leakage in dengue - is an alternative. Chest X-rays can only detect significant pleural effusions and, therefore, ultrasonography of the chest and abdomen may be preferential. The main advantages of ultrasonography are its high sensitivity to detect small amounts of pleural effusion and ascites [7], [8], [9], [10], [11], [12] and the possibility to visualize the gallbladder wall, which is frequently thickened in dengue [13], [14]. So far, most dengue ultrasonography studies are cross-sectional in nature, whereas significant changes in vascular permeability may occur after the initial presentation of the patient. Serial ultrasonography may therefore be needed to detect the presence of plasma leakage. Despite the advantages of ultrasonography to detect plasma leakage in dengue, the clinical application is still limited in resource poor settings, because it requires sophisticated facilities and expertise. Lately, more affordable handheld ultrasound devices have become available, which can be used at the bedside.

The aim of our study was to determine the predictive diagnostic value of serial ultrasonography for severe dengue. Measurements were done using a handheld ultrasound device at the bedside. We compared the ability to detect plasma leakage by serial handheld ultrasonography with conventional ultrasonography performed by a radiologist. The presence of plasma leakage detected by handheld ultrasonography was compared with frequently used clinical and laboratory parameters for plasma leakage and related to severe dengue as a clinical outcome.

## Methods

### Patients and study design

For this prospective observational study, we included patients with dengue infection, aged 14 years or above, who were admitted to Rumah Sakit Hasan Sadikin, an academic referral hospital in Bandung West Java, Indonesia, from March 2011 to January 2012. All patients had a clinically suspected dengue infection, which was retrospectively classified as non-severe or severe dengue according to WHO criteria from the guidelines edition 2009 (table 1) [4]. According to these guidelines a narrow pulse pressure (difference between systolic and diastolic blood pressure of 20 mmHg or less) is also a sign of shock. The presence of plasma leakage was confirmed by a significant hematocrit change (minimal 20%) and/or single high hematocrit values (>50 and >44% for men and women respectively), and/or by plasma leakage seen during ultrasonography. In case of a discrepancy in the presence or absence of plasma leakage between handheld or conventional ultrasonography, the latter was used as a golden standard. All patients had to be enrolled either in the febrile phase (defined as a temperature of 37.5°C or more on that day) or in the critical phase (defined as the period within 48 hours after defervescence and before platelet counts increased again) of dengue infection. Patients with concurrent chronic disease and pregnant patients were excluded.

Demographic, clinical, laboratory and ultrasonography data were collected using a standardized data collection form. Ultrasonography was not part of routine clinical care. A full blood count, including hematocrit measurements, was daily performed as part of routine clinical care, until platelet counts had shown a substantial increase and the patient had improved clinically. Patients were asked to return 2–3 weeks after discharge for follow up, which included clinical evaluation, blood drawing for full blood count including hematocrit for baseline values, serology and handheld ultrasonography. The degree of hemoconcentration was calculated according to the formula; % hemoconcentration = [(peak hematocrit surrounding defervescence)-(convalescent hematocrit or minimum hematocrit during admission)]/(convalescent hematocrit or minimum hematocrit during admission)×100.

### Laboratory diagnosis

Samples of dengue suspected cases were tested for presence of viral RNA by reverse-transcriptase PCR and samples were tested for dengue specific IgM and IgG (Panbio, Windsor, Australia), interpreted according to the manufacturer's instructions. Samples that were IgM positive, had an IgM or IgG conversion, a 4-fold titer increase in IgG and/or IgG levels comparable to a HI titer of at least 1∶2560 (the IgG cut off point set by the manufacturer to detect secondary infection) were considered dengue positive. Patients with both negative PCR and dengue serology results were excluded.

### Ultrasonography procedures

Bedside ultrasound examinations were performed daily, using a handheld portable ultrasound device (Signos personal ultrasound) with a 3.5 MHz transducer. Measurements started on the day of study enrollment until the day of discharge. All handheld ultrasound examinations were carried out by study physicians, who first received a four-week practical training in systematically detecting ascites, pleural effusion and determining the gallbladder wall aspect and thickness. To validate the results of these portable ultrasonograms, one conventional ultrasound was made per patient by a radiologist. This was done in the critical phase, since the likelihood of finding plasma leakage is the highest at this time-point. Study physicians and radiologists were blinded for each other's results.

**Figure 1:**
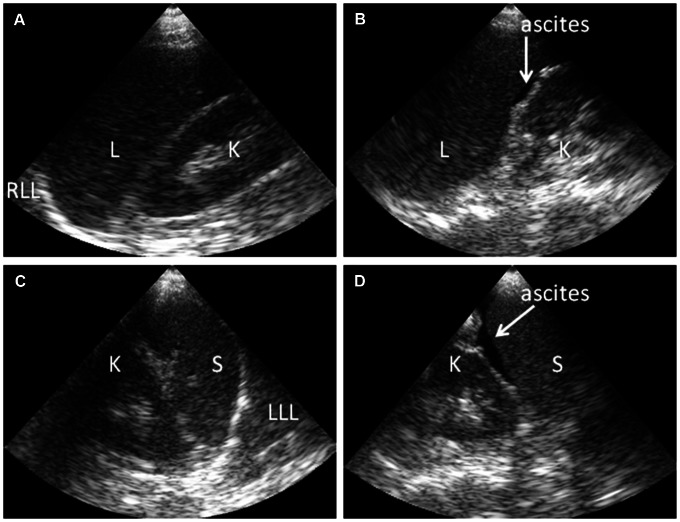
Ultrasonography findings showing presence or absence of ascites in dengue patients. Handheld ultrasonography performed at the bedside with the patient in supine position during examination. (A) Normal hepatic-renal area. (B) Minimal ascites in hepato-renal area (arrow). (C) Normal spleno-renal area. (D) Ascites in spleno-renal area (arrow). Abbreviations: L: liver; K: kidney, RLL: right lower lobe of lung; LLL: left lower lobe of lung; S: Spleen.

**Figure 2:**
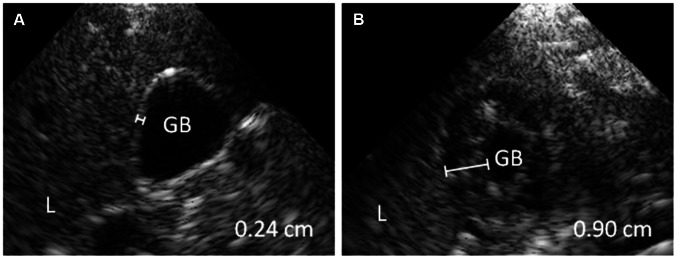
Ultrasonography findings showing a normal and thickened gallbladder wall in dengue patients. Handheld ultrasonography performed at the bedside with the patient in supine position during examination. (A) normal gallbladder wall (<0.30 cm) (B) and thickened gallbladder wall. The gall bladder wall is thickened with 0.90 cm and has a subserosal fluid layer, giving the gallbladder wall a multiple layer aspect. Abbreviations: L: liver; GB: gallbladder lumen.

**Figure 3:**
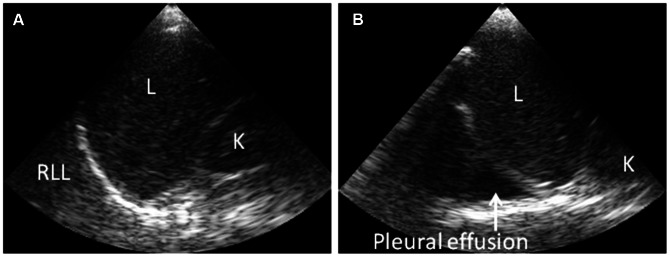
Ultrasonography findings showing presence or absence of pleural effusion in dengue patients. Handheld ultrasonography at the bedside with patient in supine position during examination. (A) Normal right costophrenic angle. (B) Pleural effusion in the right costophrenic angle (arrow). Abbreviations: L: liver; K: kidney, RLL: right lower lobe of lung.

Ultrasonography was performed after a minimum of 6 hours of fasting, with the patient in supine position. The abdomen was screened for the presence or absence of ascites in the hepato-renal pouch and spleno-renal area (figure 1 A–D). The conventional ultrasonography by the radiologist additionally screened for free fluid around the urine bladder, which was not feasible with the portable ultrasound device. Presence of multiple layers in the gallbladder wall and/or a gallbladder wall thickness of 0.3 cm or more were considered as a thickened gallbladder wall (figure 2 A and B). The presence or absence of pleural effusion was determined by visualizing bilateral costophrenic angles in supine position (figure 3 A and B).

### Ethics

The Study Research Ethics Committee of the Faculty of Medicine of the Padjadjaran University, Bandung, Indonesia, approved all legal, ethical, radiological aspects of the study. Written informed consent was obtained from all patients or from their parents, in case the patient was below 17 years of age. All patients were able to provide informed consent.

### Data presentation and statistical analysis

Data are expressed as medians with interquartile ranges (IQR) or numbers with percentages. Differences in non-continuous data of 2 groups were analyzed by Pearson's Chi-square test or by Fisher's exact test in case of expected counts <5. Continuous variables between 2 groups were analyzed by unpaired t-test in case of normally distributed data; and by Mann Whitney U test in case of non-parametric data. Relationships between continuous data were examined by Pearson's correlation for parametric data and Spearman's correlation for non-parametric data. A p-value<0.05 was considered significant. All analysis were performed using SPSS (version 18.0) statistical program.

## Results

### Clinical characteristics

Among the 71 patients with clinically suspected dengue infection, 66 patients had a positive PCR and/or serology and were included. Fifty-five (83%) patients were retrospectively classified as non-severe dengue and 11 (17%) as severe dengue according to the 2009 WHO classification [4]. All patients with severe dengue had plasma leakage with vascular collapse; none of the patients had severe bleeding or organ failure (table 1). Patient characteristics and baseline data are presented in table 2. The first day of fever was defined as the first day of illness. All patients were discharged from the hospital in good health.

**Table 2:** Patient characteristics and baseline data for non-severe and severe dengue patients.

	Non-severe dengue	Severe dengue
	n = 55 (83%)	n = 11 (17%)
Age; years	22 (19–30)	19 (15–29)
Male sex; n (%)	32 (58)	6 (55)
Duration of illness; days	6 (5–7)	6 (5–7)
Phase of dengue		
Febrile phase; n (%)	16 (29)	2 (18)
Critical phase; n (%)	39 (71)	9 (82)
Abdominal pain; n (%)	21 (38)	6 (55)
Nausea; n (%)	35 (64)	5 (46)
Vomiting; n (%)	8 (15)	3 (27)
Blood pressu; mmHg		
Systolic	110 (100–120)	100 (90–100)**
Diastolic	75 (70–80)	70 (60–80)
Pulse pressure; mmHg	40 (30–40)	20 (20–30)***
Pulse rate; per minute	84 (78–92)	92 (72–108)
Respiratory rate; per minute	22 (20–24)	20 (20–24)
Temperature; °C	36.6 (36.2–37.3)	36.1 (35.3–38.1)
Hemoglobine; g/dL	13.9 (13.0–15.5)	13.2 (11.1–16.2)
Hematocrit; %	41 (37–45)	39 (35–47)
Platelet count; ×10^9^/L	60 (34–83)	31 (20–54)*
Leukocyte count; ×10^3^/mL	3.6 (2.6–5.0)	3.8 (3.2–5.3)
Albumin; g/dL	3.8 (3.6–4.1)	3.7 (3.1–3.8)
ALAT; U/dL	30 (18–77)	46 (35–69)
Creatinin; mg/dL	0.8 (0.6–0.8)	0.7 (0.6–0.8)

* p<0.05.

** p<0.01.

*** p<0.001.

Data are represented in medians (interquartile range) for continuous data or in numbers (percentage) (n (%)) for non-continuous data. Comparisons of non-continuous and continuous data between non-severe and severe dengue patients were analyzed by Pearson's Chi Square test/Fisher's exact test and Mann-Whitney U test, respectively. P value<0.05 was considered significant. Abbreviations: ALAT: alanine aminotransferase.

### Subclinical plasma leakage detected by handheld ultrasonography

At enrollment, ascites and/or pleural effusion were found by handheld ultrasonography in 11/55 (20%) patients in the non-severe dengue group and in 6/11 (55%) patients of the severe dengue group (p = 0.03) (table 3). As 6 of 17 patients with ascites or pleural effusion at enrollment developed severe dengue, the positive predictive value (PPV) of detection of plasma leakage by handheld ultrasonography for development of severe dengue was 35%. None of the patients had clinical signs or symptoms suggestive for plasma leakage at enrollment, as determined by careful history taking and physical examination by the study clinicians. Four of the 11 patients eventually classified as having severe dengue, already fulfilled criteria for severe dengue at the time of enrollment due to subclinical plasma leakage combined with a narrow pulse pressure. Five of the 49 patients without ultrasonographic evidence of plasma leakage at enrollment, eventually developed severe dengue, yielding a negative predictive value (NPV) of 90%. Two of these patients already had a narrow pulse pressure at enrollment without further clinical evidence for plasma leakage.

**Table 3:** Ultrasonography findings of plasma leakage and hemoconcentration in non-severe and severe dengue patients.

	Non-severe dengue	Severe dengue
	n = 55 (83%)	n = 11 (17%)
**Portable ultrasonography enrollment**		
Pleural effusion only; n (%)	2 (4)	0 (0)
Ascites only; n (%)	6 (11)	5 (45)*
Pleural effusion and ascites; n (%)	3 (5)	1 (9)
Total: Plasma leakage at enrollment; n (%)	11 (20)	6 (55)*
Gallbladder wall thickened; n (%)	34 (65)^3^	9 (90)^1^
Gallbladder wall thickness; cm	0.36 (0.27–0.50)^6^	0.54 (0.34–0.75)^1^ ***
**Portable ultrasonography follow up**		
New pleural effusion only; n (%)	1 (2)	0 (0)
New ascites only; n (%)	4 (7)	1 (9)
New pleural effusion and ascites; n (%)	1 (2)	3 (27)*
Total: New plasma leakage in follow up; n (%)	6 (11)	4 (36)
Total: Plasma leakage at any time; n (%)	17 (31)	10 (91)***
New gallbladder wall thickening in follow up; n (%)	3 (5)	2 (18)
Total: Gallbladder wall thickened at any time; n (%)	37 (67)	11 (100)
**Hemoconcentration and bleeding follow up**		
Hemoconcentration minimal 20%; n (%)	12 (23)^3^	9 (90)^1^ ***
High hematocrit value; n (%)	3 (6)^3^	1 (10)^1^
Spontaneous bleeding; n (%)		
None	38 (69)	6 (55)
Epistaxis	10 (18)	3 (27)
Gingival bleeding	7 (13)	1 (9)
Menorrhaghia	0 (0)	1 (9)

1 2 3 or ^6^ = the number of observations missing.

* p<0.05.

*** p<0.001.

Data are represented in medians (interquartile range) for continuous data or numbers (percentage) (n (%)) for non-continuous data. Comparisons of non-continuous and continuous data between non-severe and severe dengue patients were analyzed by Fisher's exact test and Mann-Whitney U test, respectively. P value<0.05 was considered significant.

After admission, another 10 patients developed ultrasonographic findings of plasma leakage, mainly in the critical phase (80%). In total, 17/55 (31%) patients in the non-severe and 10/11 (91%) in the severe dengue group developed ultrasonographic evidence of plasma leakage (p<0.001) (table 3). The diagnosis of plasma leakage in the patient with severe dengue without ultrasonographic evidence of plasma leakage was based on an >20% increase in hematocrit values. Moreover, three patients without evidence of plasma leakage during follow up developed hypotension and/or a narrow pulse pressure. In 9/11 (82%) severe dengue cases, shock developed without preceding clinical symptoms or signs of plasma leakage. The remaining two patients had symptomatic pleural effusions. Ascites was more frequently detected by ultrasonography than pleural effusion, which was found either right-sided or bilateral (table 3).

### Gallbladder wall thickening

A thickened gallbladder wall was found in 34/52 (65%) of non-severe and 9/10 (90%) of severe dengue patients at enrollment (p = 0.15) (table 3). The median thickness of the gallbladder wall at enrollment was 0.36 cm (IQR, 0.27–0.50 cm) in patients with non-severe dengue versus 0.54 cm (IQR, 0.34–0.75 cm) in those with severe dengue (p<0.01). Gallbladder wall thickness could not be determined in six patients at enrollment, because these patients had taken food. Eventually, a thickened gallbladder wall was detected in 37/55 (67%) of the non-severe dengue and 11/11 (100%) of the severe dengue patients (p = 0.27). Detection of a thickened gallbladder wall at enrollment had a PPV of 21% and a NPV of 91% for severe dengue. In patients who developed ascites or pleural effusion, thickening of the gallbladder preceded the detection of ascites or pleural effusion by 1 to 3 days in 6/10 (60%) of the non-severe and in 6/9 (67%) of the severe patients. Multiple layers were frequently visible in thickened gallbladder walls suggesting the presence of a sub-serosal fluid layer (figure 2 B).

Patients with subclinical plasma leakage at enrollment had a thicker gallbladder wall than those without plasma leakage with median values of 0.59 cm (IQR, 0.38–0.79 cm) and 0.35 cm (IQR, 0.27–0.44 cm) respectively (p<0.01). There was an inverse correlation of gallbladder wall thickness with platelets counts at enrollment (r_s_ = −0.53, p<0.001). In contrast, no significant correlation existed with clinical symptoms (nausea, vomiting or abdominal pain) and laboratory values (absolute value of hematocrit or its change in time, serum albumin, alanine aminotransferase (ALAT)) (data not shown).

Regarding the timing of the ultrasonographic abnormalities in relation to the day of illness, in patients developing pleural effusion or ascites and gallbladder wall thickening during the study, these findings were already detectable on the first ultrasonography performed at enrollment in 17/27 (63%) and 43/48 (90%) of patients, respectively. The first ultrasonography was performed after a median duration of 6 days of illness (IQR, 5–7 days). The median day plasma leakage was first detected by ultrasound was day 7 of illness (IQR, 6–7 days). The maximum gallbladder wall thickness was also observed on this day (IQR, 6–8).

### Comparing serial handheld ultrasonography with conventional ultrasonography

Fifty-five conventional ultrasound reports were available to serve as the golden standard for the detection of plasma leakage by serial handheld ultrasonography. In 48/55 patients (87%), the handheld ultrasound scored the same results for the presence or absence of plasma leakage as the conventional ultrasound on that same day. In an additional 4/55 patients (7%) portable ultrasonography missed the presence of plasma leakage on the same day, but still detected plasma leakage one day after the conventional ultrasound. In 3/55 patients (5%), handheld ultrasonography failed to detect plasma leakage seen by conventional ultrasonography. This was partly because conventional ultrasonography also screened for ascites in the pelvis. Gallbladder wall thickness measurements were available for 45 patients using the conventional ultrasonography. For 37/45 (82%) of the handheld ultrasonography measurements, the presence or absence of gallbladder wall thickening was identical to the conventional ultrasonography. There were no cases in which conventional ultrasonography did not show plasma leakage while handheld ultrasonography on the same day did.

### Hematocrit is a poor indicator of plasma leakage compared to handheld ultrasonography

**Figure 4:**
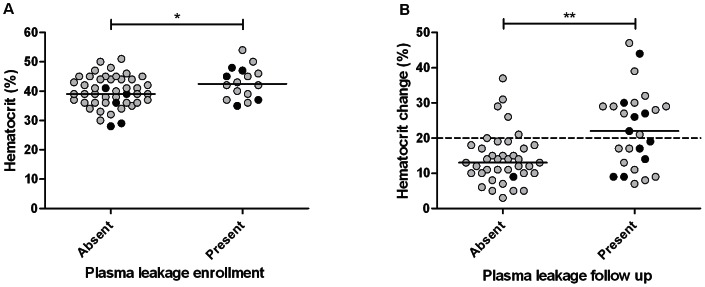
Hematocrit values in dengue patients with and without subclinical plasma leakage detected by handheld ultrasonography. (A) Absolute hematocrit values (%) (Normal maximum hematocrit value 50% and 44% for men and women, respectively) related to the presence or absence of plasma leakage detected by handheld ultrasonography on the day of enrollment. (B) Changes in hematocrit (%) related to the presence or absence of plasma leakage during the follow up of adult Indonesian dengue patients. Plasma leakage was detected in the form of ascites or pleural effusion by daily handheld ultrasonography at the bedside performed by a clinician and by conventional ultrasonography. The black dots indicate patients who developed severe dengue characterized by shock. The horizontal continuous lines represent median values. The horizontal interrupted line represents the reference value for hematocrit change (normally <20% change) P-values were determined by unpaired t-test for hematocrit values and by Mann Whitney U test for hematocrit changes. * p value<0.05; ** p value<0.01.

An elevated hematocrit value was found in only 4/66 (6%) of patients at enrollment. Single hematocrit values at enrollment were not helpful in discriminating between patients with and without ultrasonographic plasma leakage and between patients classified as non-severe or severe dengue (figure 4 A). The change in hematocrit values was also not helpful for this purpose (figure 4 B). In 13/21 (62%) of the patients in whom hemoconcentration was observed, this was only first detected after the critical phase when patients already started to recover. Other variables showed larger differences between patients with and without ultrasonographic plasma leakage. Patients with plasma leakage had lower median platelets counts (21×10^9^/L vs. 70×10^9^/L; p<0.001), serum albumin levels (36 g/L vs. 40 g/L; p<0.01) and higher ALAT levels (60 U/L vs. 26 U/L; p<0.05).

## Discussion

This study shows that serial handheld ultrasonography at the bedside may be useful to identify patients at risk for severe dengue. More than 1 out of 3 patients (35%) with ultrasonographic evidence of subclinical plasma leakage at enrollment developed shock, in contrast to only 1 out of 10 patients (10%) without subclinical plasma leakage. Dengue-related shock is known to develop rapidly and it is currently not possible to identify those at risk for shock at an early stage of illness. Our findings suggest that careful monitoring of circulatory status is merited in those patients with ultrasonographic evidence of subclinical plasma leakage since there is a significant risk for progressing to shock. Despite much research effort, reliable biomarkers for predicting severe dengue have not yet been identified. Therefore, even though the positive predictive value of ultrasonography for predicting severe dengue in our study was still far from optimal (35%), serial ultrasonography may be a considerable advance in the clinical management of dengue patients.

The high sensitivity of handheld ultrasonography to detect plasma leakage has implications for the interpretation of WHO classification of dengue severity from 1997, which continues to be used frequently in research settings [6]. Ultrasonography findings of subclinical plasma leakage changed the diagnosis from non-severe dengue (dengue fever) to severe dengue (dengue hemorrhagic fever) in 9 cases of our study population if WHO 1997 guidelines had been applied. This is in line with recent studies that report subclinical plasma leakage in patients with clinically mild dengue infection [15], [16]. Moreover, it contradicts the long-existing dichotomous view of dengue severity that is reflected in WHO classifications, in which plasma leakage is a hallmark of severe dengue infection only [8], [9]. Also in the WHO 2009 guidelines, plasma leakage remains the main criteria for severe dengue, since the other two criteria – severe bleeding and severe organ failure- are relatively rare.

Handheld abdominal ultrasonography showed a thickened gallbladder wall in 65% of the dengue cases at enrollment, and the degree of thickening was associated with dengue severity. Gallbladder wall thickening often preceded the first detection of pleural effusion or ascites and may therefore be an useful early warning sign for plasma leakage, as has also been suggested by others [7], [8], [13], . Several case reports have described the occurrence of acalculous cholecystitis in dengue patients. In most of these cases, the diagnosis was based on an ultrasonographic demonstration of a thickened gallbladder wall. Some of these patients underwent cholecystectomy whereby histology showed congestion of the serosa with presence of mononuclear cell infiltrates and lymphocyte follicle formation [18], [19]. We speculate that plasma leakage plays an important role in the pathogenesis of gallbladder wall thickening in dengue. Both ascites and gallbladder wall thickening appear and resolve around the same time and within a short time span [9], [14]. In those gallbladders with the most outspoken wall thickening, multiple layers were visible due to sub-serosal edema, suggesting plasma leakage inside the gallbladder wall. Finally, gallbladder wall thickening was not associated with abdominal pain in our patients, which makes inflammation less likely.

We demonstrated that the sensitivity for detecting plasma leakage and a thickened gallbladder wall of handheld ultrasonography performed by a non-radiologist, was comparable to conventional ultrasonography by a radiologist. Compared to ultrasonography, hematocrit proved to be a poor indicator of subclinical plasma leakage and changes in the hematocrit often occurred too late to be of clinical benefit.

There are only few prospective ultrasonography studies in dengue [9], [16], [17] and our study is, to our knowledge, the first to employ a handheld ultrasound device operated by clinicians instead of radiologists. Heterogeneity in study populations, dengue classification, timing of ultrasonography and used definitions of gallbladder wall thickening and dengue classifications complicate a proper comparison between results of previous ultrasonography studies and ours. The reported proportion of dengue patients with gallbladder thickening in earlier studies varied from 28% to 100%, while pleural effusion and ascites was found in 32% to 100% and 15% to 96%, respectively [7]–[9], [11], [12], [17]. So far, the clinical application of ultrasonography in dengue was hampered by financial and logistic constraints, but the recent availability of more affordable handheld ultrasound devices may help to overcome these restrictions. Serial bedside ultrasonography by clinicians may not only have a prognostic value by identifying dengue patients at risk for disease, but also a diagnostic value. The clinical features of dengue overlap with other endemic febrile illnesses, such as rickettsiosis and typhoid fever. The ultrasonographic detection of ascites, pleural effusion or gallbladder edema in a patient with a clinical picture of dengue supports the diagnosis of dengue [20]. Moreover, handheld ultrasonography might especially proof useful for triage during dengue outbreaks when health care facilities are overloaded.

Our study has several limitations. First, our study population consisted of patients admitted to an academic referral hospital in whom severe disease was common. This explains why plasma leakage was already visible by ultrasonography in many patients at the time of enrollment. Moreover, it makes it impossible to determine the day of illness at which plasma leakage could have been detected initially. Studies in patients presenting at an earlier stage of dengue are required to answer this question. Second, adiposity was rare in our Indonesian study population and the diagnostic performance of handheld ultrasonography in populations with more overweight may be lower. Finally, despite the fasting instructions before ultrasonography, we cannot exclude that some patients had eaten before the examination which may have resulted in an over-estimation of gallbladder wall thickness.

In conclusion, serial handheld ultrasonography holds promise as a diagnostic and prognostic tool in dengue. Compared to serial hematocrit values, serial ultrasonography had a better predictive value in identification of those patients at risk for severe dengue. Moreover, it may improve monitoring of circulatory status and enable timely adjustments in fluid balance to avoid hypovolemic shock or iatrogenic fluid overload.
